# Pharmacokinetics and exploratory efficacy biomarkers of bococizumab, an anti-PCSK9 monoclonal antibody, in hypercholesterolemic Japanese subjects 

**DOI:** 10.5414/CP203418

**Published:** 2019-09-24

**Authors:** Koutaro Yokote, Akiyuki Suzuki, Yinhua Li, Nobushige Matsuoka, Tamio Teramoto

**Affiliations:** 1 Department of Endocrinology, Hematology and Gerontology, Chiba University Graduate School of Medicine, Chiba city, Chiba,; 2 Development Japan, Pfizer R&D Japan, Tokyo, and; 3 Teikyo Academic Research Center, Teikyo University, Tokyo, Japan

**Keywords:** bococizumab, Japanese, PCSK9 inhibitor, pharmacokinetics, biomarker

## Abstract

Objective: Bococizumab, a monoclonal antibody targeting proprotein convertase subtilisin/kexin type 9, has been shown to reduce low-density lipoprotein cholesterol (LDL-C). Here, we describe the pharmacokinetics and pharmacodynamics of bococizumab and its effect on lipoprotein particle composition and other biomarkers, based on a double-blind, placebo-controlled, randomized, dose-ranging study. Materials and methods: The study consisted of two populations: Japanese subjects with uncontrolled LDL-C (LDL-C ≥ 100 mg/dL) despite treatment with atorvastatin (n = 121) and Japanese subjects naïve to lipid-lowering agents with LDL-C ≥ 130 mg/dL (n = 97). Subjects were randomized to receive either bococizumab 50, 100, or 150 mg or placebo, every 2 weeks. One arm of subjects in the atorvastatin-treated population received ezetimibe 10 mg instead of bococizumab. Results: In both populations, bococizumab exposure increased with increasing dose, and subjects with lower body weights tended to have higher exposures. Bococizumab treatment was associated with a dose-dependent reduction in LDL particles and a small increase in total high-density lipoprotein (HDL) particles. Significant reductions in lipoprotein-associated phospholipase A2 (Lp-PLA2) were observed for bococizumab-treated subjects but not for subjects treated with placebo or ezetimibe. Conclusion: Increased bococizumab dosage resulted in increased exposure. Levels of LDL and HDL particles and biomarkers such as Lp-PLA2 were also altered with bococizumab treatment. (ClinicalTrials.gov identifier: NCT02055976).

**What is known about this subject**

- Elevated levels of low-density lipoprotein cholesterol (LDL-C) have been correlated with an increased risk of cardiovascular disease.
- Inhibition of PCSK9 has emerged as a effective means of reducing serum LDL-C levels.
- Bococizumab is an investigational PCSK9 inhibitor that has been shown to reduce LDL-C levels in hypercholesterolemic subjects, when administered alone or in combination with other lipid-lowering treatments.

**What this study adds**

- Increasing doses of bococizumab resulted in increased bococizumab exposure, in both atorvastatin-treated and treatment-naïve Japanese subjects; higher exposures were observed in subjects with lower body weights.
- A dose-dependent reduction in LDL particles and an increase in total high-density lipoprotein particles were observed in Japanese subjects following bococizumab treatment; these changes were not observed in subjects treated with placebo
- Japanese subjects treated with bococizumab also exhibited reduced levels (relative to baseline) of lipoprotein-associated phospholipase A2 (Lp-PLA2).

## Introduction 

Epidemiologic studies have demonstrated a strong correlation between hypercholesterolemia, especially elevated levels of low-density lipoprotein cholesterol (LDL-C), and the risk of cardiovascular disease [1, 2]. Statins and ezetimibe are among the most widely prescribed treatments for lowering LDL-C levels, which has been shown to reduce the risk of heart attack and stroke [3, 4]. However, many patients are unable to achieve optimal levels of LDL-C despite treatment with statins and are still at risk of cardiovascular events [5, 6]. 

Serum LDL-C levels can also be effectively regulated by modulating the cellular uptake of LDL particles, which is mediated by the low-density lipoprotein receptor (LDLR). Proprotein convertase subtilisin/kexin type 9 (PCSK9), a member of the subtilisin family of kexin-like proconvertases, binds to the LDLR and targets it for degradation, resulting in reduced cellular uptake of LDL-C and, consequently, higher serum levels of LDL-C [7]. Bococizumab (RN316, PF-04950615) is a humanized monoclonal antibody that targets PCSK9 and binds with high affinity to the LDLR-binding domain of PCSK9 [8]. By binding PCSK9, bococizumab is thought to prevent the enzyme from interacting with the LDLR, thereby increasing LDLR levels and LDL particle uptake, leading to a reduction in serum LDL-C levels. 

Phase I and phase IIa studies demonstrated that bococizumab treatment was well tolerated and led to sizeable reductions in LDL-C levels when administered as either single or multiple doses, both alone and in combination with current lipid-lowering agents [9, 10]. We recently reported that, in a phase II study with Japanese subjects, bococizumab was well tolerated and significantly reduced fasting LDL-C in hypercholesterolemic subjects who were either naïve to treatment with lipid-lowering agents or were currently receiving atorvastatin [11]. Although LDL-C levels are a well-established measure of cardiovascular disease risk, there is growing recognition that LDL particle (LDL-P) levels may also be useful biomarkers for the prediction of cardiovascular events [12, 13]. For patients with discordant levels of LDL-C and LDL-P, levels of LDL-P were more strongly correlated with the risk of cardiovascular events than LDL-C [14]. Another biomarker of interest is lipoprotein-associated phospholipase A2 (Lp-PLA2), an enzyme produced by inflammatory cells in atherosclerotic plaques that has been associated with the risk of coronary heart disease [15]. In the blood, Lp-PLA2 is associated predominantly with LDL [16] and given the effect of bococizumab on levels of LDL-C, it is thought that Lp-PLA2 levels may also be affected by bococizumab administration. While the efficacy and safety of bococizumab and other PCSK9 inhibitors have been well characterized, relatively few studies [17, 18, 19, 20, 21] have focused on the pharmacokinetics (PK) and the pharmacodynamics (PD) of these molecules. The specific objectives of the current analysis were to evaluate the PK and PD of bococizumab in atorvastatin-treated and treatment-naïve subjects and to assess the effect of bococizumab on LDL-P levels and Lp-PLA2. 

On November 1, 2016, Pfizer decided to terminate further development of bococizumab as an anti-hypercholesterolemic drug, due in part to the high level of immunogenicity and the wide variation in LDL-C reduction observed in subjects from six multinational trials [22]. Specifically, a large proportion of subjects in these trials developed antidrug antibodies to bococizumab, which is a humanized monoclonal antibody [22]. These antidrug antibodies were associated with an attenuation of LDL-C reduction [22]. In contrast, little immunogenicity was observed in subjects receiving the PCSK9 inhibitors evolocumab or alirocumab, both of which are fully human monoclonal antibodies [23]. Further, both evolocumab and alirocumab were associated with sustained reductions in levels of LDL-C following long-term treatment [24, 25]. Despite the decision to terminate development of bococizumab, PCSK9 continues to be an important target for lipid-lowering drugs, and studies of bococizumab may prove helpful for other PCSK9 inhibitors. For instance, the large variation in cholesterol reduction observed among subjects who received bococizumab [22] suggests that other PCSK9 inhibitors may also need to be examined for the same effect. We believe that the findings reported in this manuscript may prove useful for understanding the mechanisms that underpin the reduction of LDL-C and cardiovascular events by PCSK9 inhibitors and provide important information on the PK properties of one of these agents. This information may assist with the development of new agents for the treatment of dyslipidemia. 

## Materials and methods 

### Subjects 

Men and women aged ≥ 20 years were enrolled in the study. Subjects whose LDL-C levels were not sufficiently controlled by a stable dose of atorvastatin (> 6 weeks before screening) as assessed using the Japan Atherosclerosis Society Guideline for prevention of atherosclerotic cardiovascular diseases [26], with fasting LDL-C ≥ 100 mg/dL and triglycerides (TG) ≤ 400 mg/dL, were enrolled into the atorvastatin-treated study population. Subjects who had not received any lipid-lowering agents, with fasting LDL-C ≥ 130 mg/dL and TG ≤ 400 mg/dL, were enrolled into the treatment-naïve study population. Further details of the inclusion and exclusion criteria were reported previously [11]. 

### Study design 

**Figure 1.**
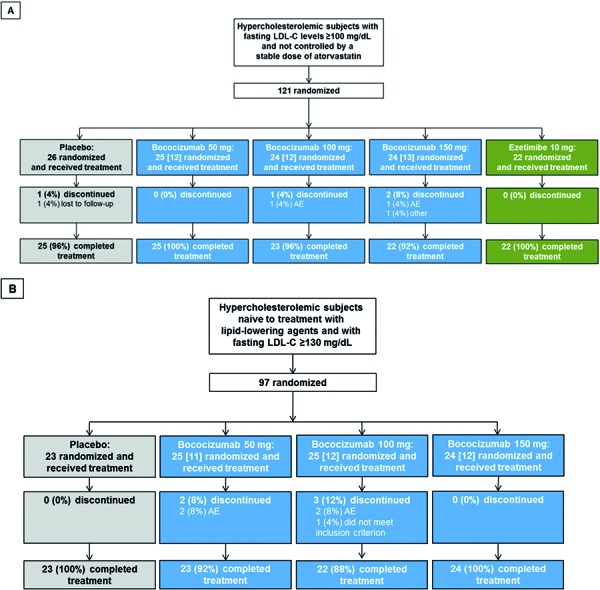
Subject disposition for the atorvastatin-treated study population (A) and the treatment-naïve study population (B). The number of subjects allocated for full PK sampling is indicated in square brackets. PK = pharmacokinetic; LDL-C = low-density lipoprotein cholesterol; AE = adverse event.

This double-blind, parallel-group, placebo-controlled, randomized, dose-ranging study was carried out in two different study populations (“atorvastatin-treated subjects” and “treatment-naïve subjects”) for 16 weeks (4 months). A total of nine parallel treatment arms with 24 subjects per arm were planned, and subjects were randomized to a treatment arm using a computer-generated randomization schedule. Atorvastatin-treated subjects were randomized 1 : 1 : 1 : 1 : 1 to receive 50 mg, 100 mg, or 150 mg bococizumab, placebo, or 10 mg ezetimibe according to the prescribed treatment schedule (Figure 1A). Treatment-naïve subjects were randomized 1 : 1 : 1 : 1 to receive 50 mg, 100 mg, or 150 mg bococizumab, or placebo according to the prescribed treatment schedule (Figure 1B). Approximately half (n = 12) of the subjects in each treatment arm (except for the ezetimibe arm) were allocated to undergo full PK sampling (Figure 1A, B). Additional details of the study design were reported previously [11]. 

The study was conducted at nine clinical sites in Japan, and the study protocol was approved by the institutional review board at each site. The study was conducted in accordance with the Declaration of Helsinki and the Good Clinical Practice guidelines. All subjects provided written informed consent before any study-specific procedures commenced. The subjects, study investigators, site staff, sponsor (excluding pre-specified, unblinded sponsor members and site personnel such as unblinded site pharmacists who prepared the drug), and sponsor members were blinded to the study drug and the lipid results (except TG). 

### Study treatment 

Subjects randomized to the bococizumab treatment arms were administered the drug subcutaneously every 2 weeks (Q14D) for the first 12 weeks. Subjects who met the dose-adjustment criteria had their dose of bococizumab adjusted on or after week 12 (Supplemental Figure S1A). The dose-adjustment criteria and the planned adjustments (Supplemental Figure S1B) were described previously [11]. All subjects in the atorvastatin-treated study population were maintained on the same dose of atorvastatin that they received at enrollment as background therapy for the duration of the study. 

### Endpoints 

The primary and secondary efficacy endpoints of this study were described previously [11]. The PK properties of bococizumab and PCSK9 levels were also evaluated. The tertiary efficacy endpoint explored in this manuscript was LDL-C composition. Investigating Lp-PLA2 was an exploratory efficacy endpoint. 

### Pharmacokinetics 

For PK analysis of bococizumab and total PCSK9, blood samples were collected from all subjects (except those in the ezetimibe arm) on days 1, 5, 8, 15, 22, 29, 36, 43, 50, 57, 71, 85, 99, 106, 113, 127, and 141. On days when subjects were to receive bococizumab (days 1, 15, 29, 43, 57, 71, and 99), blood samples were collected prior to drug administration. Subjects allocated to undergo full PK sampling had additional blood samples collected on days 2, 3, 4, 6, 7, 100, 102, 104, and 120. 

### Analytical methods 

Bococizumab and total PCSK9 concentrations in blood plasma samples were determined by ICON Laboratory Services, Inc. (formerly ICON Development Solutions, LLC Whitesboro, NY, USA). Plasma samples were stored at approximately –70 °C until analysis. Bococizumab concentrations were quantified using a validated, sensitive, and specific semi-quantitative enzyme-linked immunosorbent assay (ELISA), which had a lower limit of quantification (LLQ) of 400 ng/mL. Total PCSK9 consists of free PCSK9 and bococizumab-bound PCSK9. Total PCSK9 concentrations were quantified using a validated, sensitive, and specific electrochemiluminescence assay, which had an LLQ of 6.99 ng/mL. Lipoprotein composition and particle size were determined by nuclear magnetic resonance spectroscopy using the LipoProfile-II test at LipoScience Inc. (Raleigh, NC, USA). Lp-PLA2 concentrations in plasma samples were determined by Pacific Biomarkers, Inc. (Seattle, WA, USA). Lp-PLA2 samples were assayed using a validated, sensitive, and specific semi-quantitative ELISA method with the diaDexus PLAC^®^ test kit. 

### Statistical analyses 

The analyses of PK and biomarker data are summarized and presented descriptively. PK parameters were calculated for each subject and treatment using noncompartmental analysis of plasma concentration-time data with electronic noncompartmental analysis (eNCA, version 2.2.4), a software system developed and validated by Pfizer. For the analyses of PK parameters, subjects with full PK sampling who had at least one of the bococizumab PK parameters were included. All analys es were performed using SAS version 9.4 (SAS Institute, Cary, NC, USA). 

## Results 

### Subject disposition 

The subject disposition for the overall study population is shown in Figure 1 and was described previously [11]. Approximately half the subjects (n = 11 – 13) in each treatment group (excluding the ezetimibe group) were allocated to undergo full PK sampling. 

### Study population demographics 

The demographic and clinical characteristics for the overall study population at baseline were presented previously [11]. Subjects treated with bococizumab and who underwent full PK sampling had similar demographic characteristics (Table 1). Across the treatment arms, the mean age of subjects ranged from 53.7 to 62.2 years, and 27.3 – 83.3% of subjects were male. Similar mean (standard deviation (SD)) baseline plasma PCSK9 concentrations were observed across placebo and bococizumab-treated subjects, with values ranging from 234.3 (59.8) to 274.2 (53.2) ng/mL for the atorvastatin-treated study population and 211.1 (59.1) to 239.2 (28.3) ng/mL for the treatment-naïve study population. 

### Pharmacokinetic profile of bococizumab 

In the following section, the results of PK studies conducted in the full PK sampling population are presented, unless otherwise noted. 

**Single dose (day 1)**

Absorption of bococizumab into the systemic circulation was slow, with mean maximum plasma concentrations (C_max_) achieved within a median t_max_ of ~ 4 – 7 days post dose, for both the atorvastatin-treated and treatment-naïve study populations across all doses (Table 2) (Supplemental Figure S2). There were no marked differences between the PK profiles of the atorvastatin-treated and treatment-naïve study populations. Based on the mean values of AUC_τ_ and C_max_, bococizumab exposure increased slightly less than dose-proportionally across the 50- to 150-mg dose range in both study populations. However, this observed dose-related exposure should be interpreted with caution due to the small sample size and the large variability in bococizumab levels observed in both subject groups at all doses (Supplemental Figure S3). Based on the geometric coefficient of variation (CV), the inter-subject variability in bococizumab exposure ranged from 42 to 57% for AUC_τ_ and 42 to 61% for C_max_, with the atorvastatin-treated study population exhibiting slightly higher variability than the treatment-naïve subject population. 

**Multiple doses (day 99)**

The mean C_max_ was achieved within a median t_max_ of ~ 3 – 5 days post dose for both study populations across all dose levels (Table 2) (Supplemental Figure S2). Following attainment of C_max_, plasma concentrations appeared to decline in a mono-phasic manner, with mean terminal T_1/2 _values from ~ 8 to 11 days. The PK profiles for both study populations were generally similar across all doses. Based on the mean values of AUC_τ _and C_max_, bococizumab exposure appeared to increase greater than dose-proportionally in both study populations. Similar to the single-dose observations, this dose-related exposure should be interpreted with caution due to the large variability in bococizumab levels observed in both subject groups at all doses (Supplemental Figure S3). 

Mean accumulation ratios (R_ac_) on day 99 ranged from 1.89 to 3.18 and from 1.92 to 3.33 for the atorvastatin-treated and treatment-naïve study populations, respectively. The highest values were observed for the 150-mg dose, indicating some bococizumab accumulation at higher doses, following multiple doses every 2 weeks. The apparent clearance (CL/F) and volume of distribution (Vz/F) of bococizumab on day 99 were similar between the two study populations. The mean CL/F values ranged from 0.62 to 1.08 L/day and from 0.55 to 0.78 L/day for the atorvastatin-treated and treatment-naïve study populations, respectively (Table 2). The mean values for Vz/F ranged from 8.55 to 12.48 L and from 9.87 to 12.77 L for the atorvastatin-treated and treatment-naïve study populations, respectively (Table 2). 

The lowest mean CL/F values in each study population were observed for the groups prescribed the highest dose (150 mg) of bococizumab. For all subjects treated with bococizumab 100 mg or 150 mg (irrespective of PK sampling frequency), the minimum plasma concentrations (C_min_) observed for the atorvastatin-treated population were lower than those of the treatment-naïve study population (Supplemental Figure S4). In subjects treated with the 50-mg or 100-mg dose, steady state appears to have been reached by the third dose (day 43) for both study populations. However, for subjects treated with the 150-mg dose, bococizumab levels still had not reached steady state at day 113 (2 weeks after administration of the last dose on day 99). 

**Figure 2.**
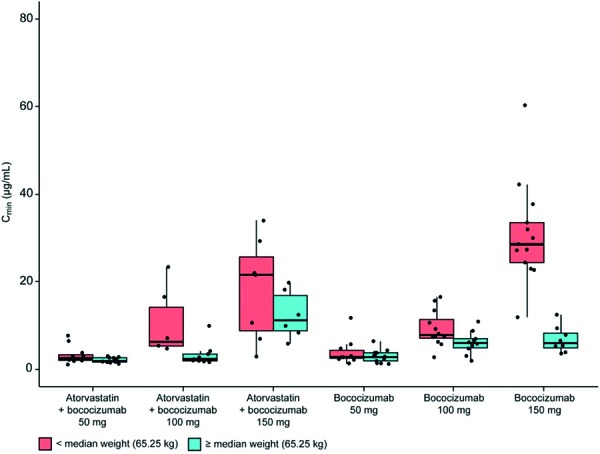
Relationship between treatment and trough level (day 99). The upper whisker extends from the hinge to the highest value that is within 1.5 × IQR of the hinge, where IQR is the inter-quartile range, or distance between the first and third quartiles. The lower whisker extends from the hinge to the lowest value within 1.5 × IQR of the hinge. Data beyond the end of the whiskers are outliers and are plotted as points. IQR = inter-quartile range.

Overall, there was substantial variability in the C_min_ of bococizumab as measured on day 99 (Figure 2). Exposure was particularly high for subjects in the treatment-naïve study population with lower body weights who were prescribed 150 mg of bococizumab. Notably, in both study populations, subjects with lower body weights tended to have higher exposures. Due to the small sample size and large variability, these data should be interpreted with caution. 

### PCSK9 analysis 

**Figure 3.**
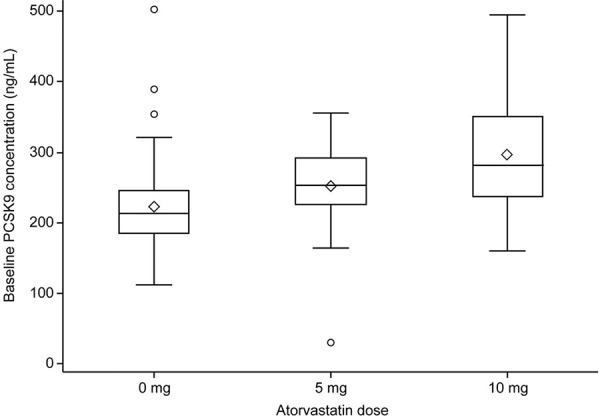
Baseline PCSK9 concentration (ng/mL) by atorvastatin dose. PCSK9 = proprotein convertase subtilisin/kexin type 9. The bottom and top of the box are the 25^th^ and 75^th^ percentiles, and the band inside the box is the median. The ends of the whiskers represent the lowest value within 1.5 × IQR of the lower quartile, and the highest value within 1.5 × IQR of the upper quartile. IQR = inter-quartile range.

The mean (SD) plasma concentration of PCSK9 at baseline for all subjects was similar across the placebo and bococizumab treatment groups within each study population. PCSK9 levels ranged from 261.2 (72.4) to 286.0 (75.8) ng/mL for the atorvastatin-treated study population and from 210.2 (48.6) to 234.4 (77.5) ng/mL for the treatment-naïve study population, as reported previously [11]. At baseline, plasma levels of PCSK9 in subjects receiving 10 mg atorvastatin were higher than those receiving 5 mg atorvastatin (Figure 3). In addition, at baseline, both atorvastatin treatment groups (5 and 10 mg) showed a trend towards higher plasma levels of PCSK9 compared with the treatment-naïve study population (0 mg atorvastatin), although a direct comparison between these subjects may not be appropriate given the differences in the enrollment criteria with respect to LDL-C levels. 

By day 85 of bococizumab treatment, the mean concentration of total PCSK9 had increased by ~ 10- to 15-fold from baseline in the atorvastatin-treated study population and by ~ 11- to 13-fold in the treatment-naïve study population (Supplemental Figure S5). For the atorvastatin-treated study population, the 100-mg dose group had the largest percent change from baseline in total PCSK9 levels (up to day 99) when compared with the 50-mg and 150-mg groups. However, this was mostly due to one atorvastatin-treated subject in the 100-mg dose group having a much lower baseline PCSK9 value (29.6 ng/mL), which resulted in a larger percent change from baseline for this group overall. When this outlier is removed, the percent change from baseline in total PCSK9 levels are similar for all three doses of bococizumab amongst the atorvastatin-treated study population. In the treatment-naïve study population, the percent change from baseline in total PCSK9 levels was also similar for all three doses of bococizumab. At all doses in both study populations, the percent change in total PCSK9 had reached a steady state by day 22; for some doses, steady state was reached by day 15. In both study populations, total PCSK9 levels at day 141 remained close to steady-state concentrations for the 150-mg groups. 

### Lipoprotein composition 

**Concentration and size of LDL-P**

**Figure 4.**
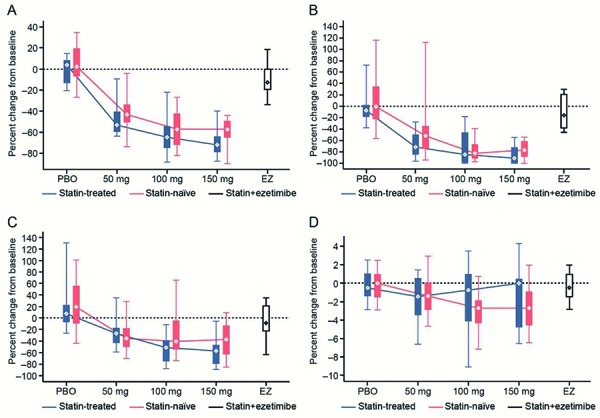
Relationship between treatment and percent change from baseline in the concentration of LDL particles (A), large LDL particles (B), and small LDL particles (C) and in LDL particle size (D) (week 12). LDL = low-density lipoprotein; PBO = placebo; EZ = ezetimibe. Whiskers represent the 5^th^ and 95^th^ percentiles, while boxes represent the 25^th^ and 75^th^ percentiles. Diamonds represent the median.

Following bococizumab treatment, a dose-response relationship was observed for the reduction of LDL-P in both study populations when compared with baseline (Figure 4) (Supplemental Table S1). A dose-response relationship was not observed for large or small LDL-P in either study population (Figure 4) (Supplemental Table S1). There were no marked changes in the concentrations of any of the LDL-P values in subjects treated with placebo (both study populations) or ezetimibe (atorvastatin-treated study population only). Following bococizumab treatment, a small decrease in LDL-P size was observed in some dose groups when compared with baseline, particularly in the treatment-naïve study population. There were no marked changes in the size of LDL-P in subjects treated with placebo (both study populations) or ezetimibe (atorvastatin-treated study population only). 

**HDL particles**

**Figure 5.**
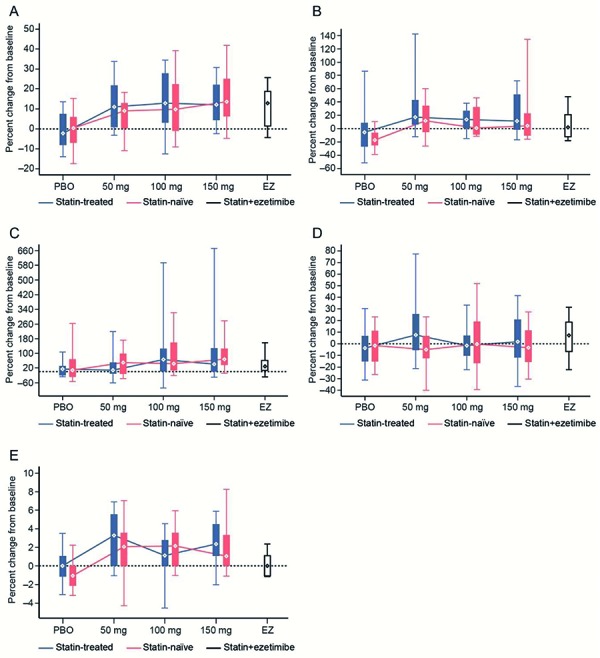
Relationship between treatment and percent change from baseline in the concentration of HDL particles (A), large HDL particles (B), medium HDL particles (C), and small HDL particles (D), and in HDL particle size (E) (week 12). HDL = high-density lipoprotein; PBO = placebo; EZ = ezetimibe. Whiskers represent the 5^th^ and 95^th^ percentiles, while boxes represent the 25^th^ and 75^th^ percentiles. Diamonds represent the median.

Following treatment with bococizumab, there was an increase in the concentration of total high-density lipoprotein (HDL) particles as well as large and medium HDL particles in both study populations when compared with baseline, but a dose-response relationship was not observed (Figure 5) (Supplemental Table S1). There were no marked changes in the concentrations of any of the HDL particles in subjects treated with placebo (both study populations) (Supplemental Table S1) but subjects treated with ezetimibe (atorvastatin-treated study population only) (Supplemental Table S1) showed an increase in the concentration of total HDL particles. There was a small increase in the size of HDL particles following bococizumab treatment when compared with baseline. In contrast, there were no marked changes in the size of HDL particles in subjects treated with placebo (both study populations) or ezetimibe (the atorvastatin-treated study population only). 

**VLDL and IDL particles**

Due to large variations observed in the concentration and size of very-low-density lipoprotein (VLDL) and intermediate-density lipoprotein (IDL) particles, it was difficult to observe any trends in these data. 

**Correlation between LDL-P concentration and lipoprotein(a)**

A post-hoc analysis of the correlation between LDL-P and lipoprotein(a) (Lp(a)) concentrations was also performed. In both study populations, subjects prescribed bococizumab were observed to have larger percent changes in Lp(a), when compared with subjects prescribed placebo (Supplemental Figures S6A, B). This resulted from the fact that Lp(a) was reduced in subjects in the bococizumab treatment arms, when compared with subjects prescribed placebo, as reported previously [11]. An analysis incorporating all three bococizumab treatment groups and the placebo group revealed a weak positive correlation between the change in LDL-P concentration and the change in Lp(a), in both study populations (Supplemental Figures S6A, B). No correlation was observed between changes in LDL-P concentration and changes in Lp(a) when analyzing only the atorvastatin-treated study population treated with bococizumab (Pearson correlation coefficient = 0.23) (Supplemental Figure S6C). Similarly, there was no correlation observed in the treatment-naïve study populations treated with bococizumab when the placebo group was excluded (Pearson correlation coefficient = 0.29) (Supplemental Figure S6D). 

### Lp-PLA2 

**Figure 6.**
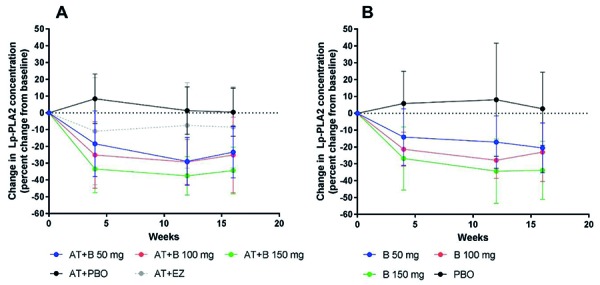
Percent change from baseline in Lp-PLA2 in the atorvastatin treatment groups (A) and the treatment-naïve groups (B). Lp-PLA2 = lipoprotein-associated phospholipase A2; AT = atorvastatin; B = bococizumab, PBO = placebo; EZ = ezetimibe. Error bars represent standard deviation.

In the atorvastatin-treated study population, treatment with bococizumab was associated with significant reductions in Lp-PLA2 at week 12 and week 16 when compared with baseline (Figure 6). The mean (SD) percent changes from baseline at week 12 in the atorvastatin-treated study population were –28.96 (14.25), –29.37 (13.45), and –37.54 (11.50) in the 50-mg, 100-mg, and 150-mg treatment groups, respectively. The corresponding values at week 16 were –23.38 (15.41), –25.17 (22.56), and –34.41 (13.90), respectively. Similarly, reductions in Lp-PLA2 were also observed in the treatment-naïve study population at week 12 and week 16 following bococizumab treatment (Figure 6). The mean (SD) percent changes from baseline in the atorvastatin-naïve study population were –17.10 (15.59), –27.91 (10.78), and –34.35 (19.08) in the 50-mg, 100-mg, and 150-mg treatment groups, respectively. The corresponding values at week 16 were –20.54 (14.74), –23.05 (17.40), and –33.89 (17.22), respectively. There were no significant changes in Lp-PLA2 in subjects treated with placebo (both study populations) or ezetimibe (atorvastatin-treated study population only). 

### Immunogenicity 

As reported previously, 50.3% (74/147) of subjects who received bococizumab were found to be positive for anti-drug antibodies (ADAs) [11]. The proportion of ADA-positive subjects was similar between the atorvastatin-treated population (46.6%) and the treatment-naïve population (54.1%), and there did not appear to be a dose-dependent relationship in either group (Supplemental Table S2). Of the 74 ADA-positive subjects, samples from 55 subjects were also tested for the presence of neutralizing antibodies (nAb). Samples from the remaining subjects were not tested, due to interference from PF-04950615 concentration and/or PCSK9. Overall, 35 of the 55 ADA-positive subjects (63.6%) who were tested were found to have positive nAb titers (Supplemental Table S2); the overall incidence of nAb positivity was at least 23.8% (35/147) across all subjects. However, as samples from 19 ADA-positive subjects were not tested in the nAb assay, the true incidence of nAb positivity may have been underestimated. The incidence of nAb positivity across the three dose groups ranged from 8.3 to 34.0% across all subjects (Supplemental Table S2). There was no apparent difference in the incidence of nAbs between the atorvastatin-treated population and the treatment-naïve population. 

As previously reported, there seemed to be no difference in bococizumab efficacy (lowering LDL-C) between subjects who were positive for ADAs compared with subjects who were negative for ADAs in this study [11]. However, as previously noted [11], one subject experienced an attenuation of the LDL-C lowering response and a reduction in the plasma concentration of bococizumab that coincided with an increase in ADA titer (Supplemental Figure S7). 

## Discussion 

This double-blind, parallel-group, placebo-controlled, randomized, dose-ranging study provides insights into the PK and PD properties of bococizumab and the effect of bococizumab treatment on lipoprotein composition in atorvastatin-treated and treatment-naïve subjects in Japan. Increasing doses of bococizumab resulted in higher bococizumab exposure in both study populations. Reductions in total LDL-P concentrations and increases in HDL particle concentrations were observed following bococizumab treatment. Significant reductions in the cardiac biomarker Lp-PLA2 were also observed for bococizumab-treated subjects. Evaluating the effects of bococizumab administration beyond its LDL-C-lowering properties is of clinical relevance given the limited information available on the PK and PD properties of PCSK9 inhibitors and the growing interest in different biomarkers for assessing the risk of cardiovascular events as well as the effects of lipid-lowering agents on these biomarkers. 

Bococizumab exposure increased with increasing dose, with the highest exposures observed for subjects treated with bococizumab 150 mg, particularly in the treatment-naïve study population. The greater than dose proportional increase of bococizumab exposure was also reported in previous phase 1 studies following administration of single and multiple doses [27]. When stratified according to body weight, subjects with lower body weights had higher exposure. This is consistent with body weight being a significant predictor of LDL response, as observed previously [28] and also discussed in the primary manuscript. Terminal T_1/2_ values following multiple doses of bococizumab were estimated to be from 8 to 11 days in this study, which are similar to the 7 to 9 days reported from the study of a single 150-mg subcutaneous dose of bococizumab administered to untreated subjects with LDL ≥ 130 mg/dL [21]. Given that the last PK sampling point after the day 1 dose was on day 15, there was no time period of sufficient length to estimate the half-life accurately in order to extrapolate AUC and calculate AUC_inf_. 

Bococizumab exposure was lower in the atorvastatin-treated study population compared with the treatment-naïve study population, for subjects treated with bococizumab 100 mg or 150 mg. The atorvastatin-treated study population had higher concentrations of plasma PCSK9 at baseline when compared with the treatment-naïve study population. Atorvastatin has been shown to increase PCSK9 expression by modulating the sterol regulatory element-binding protein-2 (SREBP-2) [29, 30]. Although the two study populations were subject to different enrollment criteria with respect to LDL-C levels, which could have contributed to the increased plasma PCSK9 levels observed in the atorvastatin-treated study population at baseline, this increase could also be reasonably attributed to atorvastatin treatment. Upregulation of PCSK9 by atorvastatin (via SREBP-2) in the atorvastatin-treated study population may have resulted in greater clearance of PCSK9-bound bococizumab, leading to reduced bococizumab exposure when compared with the treatment-naïve study population. The use of statins has been associated with increased clearance of bococizumab, as its elimination is known to be target-mediated [20]. 

Following bococizumab treatment, a clear dose-dependent reduction in the concentration of total LDL particles was observed in both the atorvastatin-treated and treatment-naïve study populations. A number of other studies have also shown that PCSK9 inhibition results in significant reductions in LDL particle concentration [31, 32]. For example, treatment with the PCSK9 inhibitor alirocumab has also been shown to reduce the concentration of LDL particles [32]. The reductions observed in this and other studies are consistent with the mode of action of PCSK9 inhibitors, which disrupt LDLR degradation, leading to an increase in LDLR concentration on the surface of hepatocytes. In this study, bococizumab treatment resulted in a small, non-significant increase in the concentration of HDL particles, similar to that which has been observed in previous studies of bococizumab [31] and alirocumab [32], which showed that PCSK9 inhibition did result in a significant increase in HDL particle concentration. Although a number of other studies [31, 32] reported a reduction in VLDL particles following PCSK9 inhibition, this was not observed in our study. Given the growing recognition of the importance of lipoprotein particles in promoting atherogenesis, the ability of PCSK9 inhibitors to modulate lipoprotein particle levels (in addition to LDL-C) may constitute an important therapeutic advance. 

While bococizumab treatment resulted in a marked reduction in Lp(a) [11], there was only a weak correlation observed between a reduction in Lp(a) and a reduction in LDL-P concentration. Furthermore, there is no correlation between the reduction in Lp(a) and the reduction in LDL-P concentration when subjects prescribed placebo are excluded from the analysis. This is not surprising given that dose dependence was observed for the reduction in LDL particles but not for Lp(a) [11]. While it is known that Lp(a) levels are reduced following treatment with PCSK9 inhibitors, the exact mechanism by which this occurs is not fully understood [33]. Our results may indicate that Lp(a) reduction following PCSK9 inhibitor treatment involves mechanisms other than uptake by upregulated LDL receptors. It is hoped that the findings of this study may shed further light on the relationship between PCSK9 inhibition and Lp(a) levels. Treatment with bococizumab also resulted in substantial reductions in Lp-PLA2 at weeks 4, 12, and 16 in both subject groups. Given that Lp-PLA2 circulates in the blood bound mostly to LDL-P [34], this observation is consistent with the observed reduction in LDL particles and with bococizumab’s mechanism of action. 

This is one of the few studies to report on the PK of a PCSK9 inhibitor and to characterize the effect of PCSK9 inhibition on lipid particle size. Another strength of this study is the fact that it is specific to the Japanese population. The weaknesses of this study include its small sample size and the fact that only half of the subjects were selected to undergo full PK sampling. Given that 19 ADA-positive subjects were not tested for nAbs due to interference by bococizumab concentration and/or PCSK9, the true incidence of nAb-positivity may have been underestimated. 

## Conclusion 

Following the administration of multiple doses of bococizumab to atorvastatin-treated and treatment-naïve subjects, PK analysis showed that drug exposure increased with increasing dose and that a steady state was reached by the third dose in subjects prescribed bococizumab 50 mg or 100 mg. In both subject groups, bococizumab treatment also led to a clear reduction in the concentration of LDL-P and Lp-PLA2, as well as a small increase in HDL particles. Although further development of bococizumab has been discontinued by the sponsor, studies of this drug may prove instructive for the future development of other humanized antibodies. Previous work on bococizumab highlights the need for long-term studies to monitor the potential emergence of antidrug antibodies and the resulting effects on efficacy, which may not be observable over a shorter duration. We also hope that the effects of PCSK9 inhibition on lipoprotein particle composition, Lp(a), and Lp-PLA2 will provide further mechanistic insights into this promising class of therapeutics. 

## Acknowledgment 

The authors wish to thank the investigators, the participating subjects, and the Pfizer Operations Group for their assistance in conducting the study. This study was sponsored by Pfizer. Editorial support for the development of this manuscript was provided by Chu Kong Liew, PhD, and Jon Edwards, PhD, of Engage Scientific Solutions and was funded by Pfizer. 

## Funding 

This study was sponsored by Pfizer. 

## Conflict of interest 

Koutaro Yokote reports research funding from Astellas; departmental sponsorship by MSD; subsidies/donations from Astellas, Boehringer Ingelheim, Bristol-Myers Squibb, Daiichi Sankyo, Sumitomo Dainippon Pharma, Eli Lilly, Kyowa Hakko Kirin, Mochida Pharmaceutical, MSD, Ono Pharmaceutical, Pfizer, Shionogi, Taisho Toyama Pharmaceutical, Takeda, Mitsubishi Tanabe Pharma, Teijin Pharma, and Toyama Kagaku Kogyo; and honoraria from Astellas, AstraZeneca, Boehringer Ingelheim, Daiichi Sankyo, Sumitomo Dainippon Pharma, Eisai, Kowa, Kowa Pharmaceutical, Kyowa Hakko Kirin, Mochida Pharmaceutical, MSD, Ono Pharmaceutical, Pfizer, Sanofi, Sanwa Kagaku Kenkyusho, Shionogi, Taisho Toyama Pharmaceutical, Takeda, and Mitsubishi Tanabe Pharma. Tamio Teramoto reports research grants and honoraria from ASKA Pharmaceutical, Astellas, Bayer, Daiichi Sankyo, Kissei, MSD, and Takeda; research grants from Kowa, Mochida Pharmaceutical, and Shionogi; and honoraria from Amgen, Kobayashi Pharmaceutical, Pfizer, and Sanofi. Akiyuki Suzuki, Yinhua Li, and Nobushige Matsuoka are employees of Pfizer. 

**Table 1:** Baseline demographics of atorvastatin-treated and treatment-naïve subjects that underwent full PK sampling.

	Atorvastatin-treated study population			Treatment-naïve study population		
Variable^a^	50 mg (n = 12)	100 mg (n = 12)	150 mg (n = 13)	50 mg (n = 11)	100 mg (n = 12)	150 mg (n = 12)
Age (years)	55.3 ± 10.4	55.3 ± 10.9	62.2 ± 6.1	58.3 ± 8.1	60.9 ± 12.2	53.7 ± 8.8
Male gender	10 (83.3)	8 (66.7)	7 (53.8)	3 (27.3)	5 (41.7)	10 (83.3)
Weight (kg)	66.1 ± 12.6	65.2 ± 10.6	63.9 ± 12.1	60.4 ± 12.7	62.4 ± 9.1	67.7 ± 17.8
BMI (kg/m^2^)	23.1 ± 3.6	24.1 ± 3.7	24.1 ± 3.1	24.3 ± 4.0	25.1 ± 2.8	24.3 ± 4.7
PCSK9 (ng/mL)	234.3 ± 59.8	252.5 ± 85.1	274.2 ± 53.2	239.2 ± 28.3	219.8 ± 46.1	211.1 ± 59.1

^a^Values are mean ± standard deviation or n (%). BMI = body mass index; PCSK9 = proprotein convertase subtilisin/kexin type 9; PK = pharmacokinetic.

**Table 2.** Summary of plasma pharmacokinetic parameters following single and multiple subcutaneous doses of bocozicumab (atorvastatin-treated and treatment-naïve study populations).

	Atorvastatin-treated study population			Treatment-naïve study population		
Parameter (units)^a^	50 mg	100 mg	150 mg	50 mg	100 mg	150 mg
Day 1 (single dose)						
N	12	12	13	11	12	12
AUCτ_,_ (µg×day/mL)	32.98 (57)	52.33 (49)	77.11 (43)	32.97 (42)	51.49 (46)	82.05 (45)
C_max_ (µg/mL)	3.173 (61)	5.074 (50)	7.382 (45)	2.994 (44)	4.744 (47)	7.726 (42)
t_max_ (day)	4.01 (3.00 – 5.96)	4.97 (3.94 – 7.00)	5.94 (1.94 – 7.00)	5.94 (3.94 – 6.95)	5.45 (2.95 – 6.94)	6.94 (2.96 – 14.0)
Day 99 (multiple dose)						
N, n	10, 10	8, 7	8, 8	10, 9	11, 11	12, 7
AUCτ (µg×day/mL)	63.54 (40)	92.46 (127)	242.5 (81)	63.74 (57)	136.6 (32)	273.5 (100)
C_max_ (µg/mL)	6.197(36)	8.343 (119)	21.91 (76)	5.874 (55)	12.22 (29)	23.64 (88)
C_min_ (µg/mL)	2.176 (65)	4.041 (147)	12.95 (94)	2.858 (64)	6.571 (40)	13.47 (139)
t_max_ (day)	3.03 (2.92 – 6.98)	2.98 (1.00 – 4.97)	2.97 (0.964 – 4.99)	2.99 (0.985 – 7.06)	2.98 (0.988 – 4.98)	4.98 (2.97 – 7.00)
T_1/2_ (day)	7.716 ± 1.759	9.471 ± 2.289	10.56 ± 1.593	9.404 ± 2.145	9.570 ± 2.234	9.333 ± 2.754
R_ac_	2.027 (47)	1.889 (60)	3.176 (56)	1.920 (35)	2.665 (46)	3.330 (57)
CL/F (L/day)	0.7870 (40)	1.082 (127)	0.6184 (81)	0.7845 (57)	0.7317 (32)	0.5490 (100)
Vz/F (L)	8.552 (48)	12.48 (107)	9.330 (94)	9.866 (65)	9.881 (39)	12.77 (45)

^a^Geometric mean (geometric %CV) for all except: median (range) for t_max_; arithmetic mean ± SD for T_1/2_. N = Number of subjects in the treatment group and contributing to the mean; n = number of subjects where T_1/2_, and Vz/F_,_ were reported. AUCτ = area under the plasma concentration-time curve over the dosage interval (τ = 2 weeks); CL/F = apparent total clearance; C_max_ = maximum concentration; C_min_ = minimum concentration; PK = pharmacokinetic; R_ac_ = accumulation ratio; T_1/2_ = terminal half-life; t_max_ = time to reach maximum concentration; Vz/F = apparent volume of distribution.

## Supplemental material

Supplemental material Supplementary Tables and Figures
