# Supplementary material for: Pharmacokinetics and exploratory efficacy biomarkers of bococizumab, an anti-PCSK9 monoclonal antibody, in hypercholesterolemic Japanese subjects
Source: Int J Clin Pharmacol Ther. 2019 Sep 24;57(12):575–89. doi: 10.5414/CP203418 (PMC6862531; doi:10.5414/CP203418)

SUPPLEMENTAL MATERIAL

Table S1. Summary of changes in lipoprotein particle concentrations in treatment groups (Week 12)

	Atorvastatin-treated study population					Treatment-naïve study population			
		Bococizumab					Bococizumab		
**Variable, mean (SD)**	**Placebo (n=26)**	**50 mg (n=25)**	**100 mg (n=24)**	**150 mg (n=23)**	**Ezetimibe (n=22)**	**Placebo (n=23)**	**50 mg (n=25)**	**100 mg (n=23)**	**150 mg (n=24)**
Total LDL particles									
Mean baseline[a]	1286.8 (217.6)	1294.6 (299.8)	1246.5 (265.4)	1235.0 (244.4)	1339.1 (296.0)	1398.4 (268.1)	1534.0 (247.1)	1455.1 (289.5)	1437.7 (255.0)
Observed mean[a]	1264.0 (209.7)	654.5 (214.1)	449.4 (231.7)	368.0 (196.5)	1184.7 (278.1)	1427.3 (246.1)	884.6 (311.7)	622.8 (252.8)	558.9 (196.5)
Mean absolute change from baseline	-22.8 (170.9)	-640.1 (306.6)	-797.1 (354.5)	-865.7 (264.2)	-154.41 (300.6)	28.9 (285.8)	-649.4 (296.7)	-843.0 (407.4)	-878.8 (312.0)
Mean percent change from baseline	-0.96 (12.4)	-48.2 (17.4)	-62.4 (21.6)	-69.5 (16.4)	-9.88 (19.5)	4.38 (19.8)	-42.2 (18.9)	-55.5 (20.8)	-60.2 (14.2)
Large LDL particles									
Mean baseline[a]	566.4 (243.0)	592.6 (181.2)	439.8 (242.0)	531.5 (220.5)	580.1 (243.5)	622.9 (251.8)	652.9 (239.4)	669.0 (262.1)	707.8 (270.0)
Observed mean[a]	522.6 (203.8)	180.9 (126.5)	105.1 (117.2)	96.35 (115.4)	513.8 (287.7)	619.0 (245.7)	304.5 (163.5)	184.3 (192.3)	183.3 (158.2)
Mean absolute change from baseline	-43.9 (136.1)	-411.8 (189.3)	-334.8 (244.7)	-439.0 (209.0)	-66.3 (201.4)	-3.8 (216.1)	-348.4 (239.8)	-520.4 (253.7)	-524.5 (210.3)
Mean percent change from baseline	49.0 (274.7)	-67.0 (23.1)	-67.3 (44.7)	-83.3 (22.8)	-9.7 (30.1)	13.4 (56.8)	-42.3 (55.7)	-74.5 (24.1)	-74.3 (18.3)
Small LDL particles									
Mean baseline[a]	590.1 (247.6)	588.1 (236.7)	711.9 (347.9)	583.8 (288.1)	654.3 (199.4)	626.2 (308.8)	724.5 (281.6)	637.8 (345.8)	603.2 (354.0)
Observed mean[a]	634.5 (227.8)	401.4 (151.2)	301.0 (177.7)	208.7 (148.3)	602.3 (189.5)	665. 6 (266.2)	485.6 (194.1)	367.4 (172.4)	313.6 (169.0)
Mean absolute change from baseline	44.4 (151.6)	-186.7 (204.6)	-411.0 (321.7)	-366.7 (282.4)	-52.1 (181.1)	39.4 (228.9)	-238.9 (235.2)	-259.3 (294.1)	-289.6 (321.0)
Mean percent change from baseline	33.9 (116.3)	-25.2 (30.8)	-52. 6 (27.1)	-56.9 (28.4)	-4.6 (29.3)	22.0 (45.3)	-28.5 (32.6)	-25.3 (46.0)	-38.6 (29.9)
LDL particle size									
Mean baseline[b]	20.9 (0.68)	20.9 (0.47)	20.6 (0.68)	20.8 (0.51)	20.8 (0.43)	20.9 (0.63)	20.9 (0.55)	21.02 (0.73)	21.1 (0.67)
Observed mean[b]	20.8 (0.59)	20.6 (0.51)	20.3 (0.46)	20.8 (0.70)	20.7 (0.46)	20.8 (0.58)	20.6 (0.47)	20.5 (0.61)	20.6 (0.56)
Mean absolute change from baseline	-0.03 (0.37)	-0.36 (0.61)	-0.34 (0.77)	-0.30 (0.68)	-0.07 (0.34)	-0.06 (0.38)	-0.25 (0.48)	-0.66 (0.48)	-0.58 (0.57)
Mean percent change from baseline	-0.10 (1.79)	-1.70 (2.8)	-1.55 (3.6)	-1.42 (3.2)	-0.32 (1.6)	-0.25 (1.81)	-1.17 (2.32)	-3.08 (2.21)	-2.70 (2.67)
Total HDL particles									

Variable, mean (SD)	Atorvastatin-treated study population					Treatment-naïve study population			
		Bococizumab					Bococizumab		
	Placebo (n=26)	50 mg (n=25)	100 mg (n=24)	150 mg (n=23)	Ezetimibe (n=22)	Placebo (n=23)	50 mg (n=25)	100 mg (n=23)	150 mg (n=24)
Mean baseline[c]	33.8 (6.1)	32.3 (4.5)	33.5 (4.3)	31.8 (2.7)	33.9 (5.1)	33.6 (6.2)	31.4 (5.3)	32.2 (5.7)	31.7 (4.6)
Observed mean[c]	33.3 (5.3)	36.3 (4.5)	38.0 (5.1)	35.8 (4.7)	37.3 (5.1)	33.0 (5.0)	33.3 (5.7)	36.8 (7.3)	36.2 (4.5)
Mean absolute change from baseline	-0.6 (3.1)	4.0 (3.9)	4.5 (4.8)	4.0 (3.2)	3.4 (3.5)	-0.7 (4.1)	1.9 (2.9)	3.9 (4.7)	4.6 (4.5)
Mean percent change from baseline	-1.0 (8.9)	13.2 (13.1)	14.3 (15.2)	13.0 (10.3)	10.6 (10.9)	-1.0 (10.4)	6.5 (9.6)	12.4 (15.1)	15.8 (16.3)
Large HDL particles									
Mean baseline[c]	6.5 (3.3)	5.5 (2.7)	6.2 (2.6)	6.4 (3.2)	6.1 (3.1)	7.2 (2.8)	5.9 (3.1)	6.6 (3.3)	7.1 (4.9)
Observed mean[c]	6.0 (3.0)	6.7 (2.8)	6.9 (3.0)	7.3 (2.9)	6.3 (2.9)	6.1 (2.4)	6.3 (2.9)	7.0 (3.0)	7.6 (4.3)
Mean absolute change from baseline	-0.5 (1.5)	1.2 (1.3)	0.7 (1.3)	0.9 (1.5)	0.1 (1.1)	-1.1 (1.0)	0.4 (1.3)	0.3 (1.1)	0.5 (1.4)
Mean percent change from baseline	-1.4 (42.9)	33.1 (46.8)	12.2 (20.5)	23.3 (31.4)	10.6 (37.8)	-16.1 (16.2)	15.0 (30.5)	9.4 (26.2)	19.6 (46.5)
Medium HDL particles									
Mean baseline[c]	7.8 (3.8)	9.9 (5.4)	6.1 (3.1)	7.3 (3.8)	8.8 (4.5)	6.8 (4.0)	6.9 (3.3)	8.1 (5.4)	6.0 (2.8)
Observed mean[c]	8.7 (4.7)	11.1 (5.0)	9.9 (5.9)	10.3 (3.4)	11.2 (5.5)	7.6 (3.6)	9.5 (4.8)	12.0 (5.7)	10.1 (3.7)
Mean absolute change from baseline	0.9 (3.1)	1.1 (6.0)	3.8 (5.2)	2.9 (4.1)	2.4 (3.1)	0.8 (3.8)	2.6 (4.0)	3.6 (3.8)	4.1 (3.0)
Mean percent change from baseline	22.6 (52.3)	34.0 (79.4)	132.2 (283.8)	105.3 (199.3)	38.6 (51.2)	89.1 (291.6)	50.7 (70.7)	88.7 (112.1)	92.3 (80.9)
Small HDL particles									
Mean baseline[c]	19.5 (4.2)	16.8 (5.2)	21.3 (4.5)	18.2 (4.4)	19.0 (4.7)	19.6 (5.8)	18.5 (3.3)	17.5 (6.5)	18.6 (4.6)
Observed mean[c]	18.5 (4.1)	18.5 (4.2)	21. 2 (5.5)	18.2 (4.6)	19.9 (5.4)	19.2 (4.9)	17.5 (4.1)	17.8 (8.0)	18.5 (6.3)
Mean absolute change from baseline	-1.0 (3.5)	1.7 (5.3)	-0.1 (3.4)	0.2 (3.8)	0.9 (3.1)	-0.4 (3.2)	-1.1 (3.3)	-0.0 (4.7)	-0.04 (3.6)
Mean percent change from baseline	-3.3 (18.3)	84. 4 (383.2)	0.6 (20.4)	3.7 (23.3)	5.2 (17.9)	2.5 (25.1)	-5.1 (18.4)	0.3 (30.1)	-1.3 (20.2)
HDL particle size									
Mean baseline[b]	9.12 (0.43)	9.03 (0.37)	9.09 (0.40)	9.14 (0.48)	9.08 (0.42)	9.22 (0.43)	9.07 (0.40)	9.17 (0.51)	9.18 (0.63)
Observed mean[b]	9.11 (0.39)	9.30 (0.35)	9.19 (0.45)	9.36 (0.40)	9.08 (0.37)	9.14 (0.37)	9.20 (0.40)	9.31 (0.47)	9.34 (0.55)
Mean absolute change from baseline	-0.00 (0.17)	0.27 (0.27)	0.10 (0.22)	0.21 (0.26)	0.00 (0.14)	-0.08 (0.17)	0.14 (0.31)	0.18 (0.23)	0.16 (0.25)
Mean percent change	-0.01 (1.82)	3.07 (3.08)	1.06 (2.46)	2.37 (2.92)	0.03 (1.56)	-0.81 (1.80)	1.56 (3.40)	2.01 (2.49)	1.82 (2.82)

Variable, mean (SD)	Atorvastatin-treated study population<br>Placebo<br>(n=26)	Bococizumab<br>50 mg<br>(n=25)	100 mg<br>(n=24)	150 mg<br>(n=23)	Ezetimibe<br>(n=22)	Treatment-naïve study population<br>Placebo<br>(n=23)	Bococizumab<br>50 mg<br>(n=25)	100 mg<br>(n=23)	150 mg<br>(n=24)
from baseline									

[a] Units = nmol/L

[b] Units = µmol/L

[c] Units = nm

HDL, high-density lipoprotein; LDL, low-density lipoprotein; SD, standard deviation.

Table S2. Immunogenicity following bococizumab administration.

Population	Bococizumab dose	N	ADA positive[a] N (%)	N[b]	nAb positive n (%[c])	nAb positive n/N (%[d])
Atorvastatin-treated study population	50 mg	25	14 (56.0)	14	8 (32.0)	8/14 (57.1)
	100 mg	24	6 (25.0)	4	3 (12.5)	3/4 (75.0)
	150 mg	24	14 (58.3)	8	3 (12.5)	3/8 (37.5)
	All doses	73	34 (46.6)	26	14 (19.2)	14/26 (53.8)
Treatment-naïve study population	50 mg	25	13 (52.0)	12	9 (36.0)	9/12 (75.0)
	100 mg	25	16 (64.0)	13	11 (44.0)	11/13 (84.6)
	150 mg	24	11 (45.8)	4	1 (4.2)	1/4 (25.0)
	All doses	74	40 (54.1)	29	21 (28.4)	21/29 (72.4)
Atorvastatin-treated and treatment-naïve study populations	50 mg	50	27 (54.0)	26	17 (34.0)	17/26 (65.4)
	100 mg	49	22 (44.9)	17	14 (28.6)	14/17 (82.3)
	150 mg	48	25 (52.1)	12	4 (8.3)	4/12 (33.3)
	All doses	147	74 (50.3)	55	35 (23.8)	35/55 (63.6)

[a] ADA negative = titer < 6.23, ADA positive = titer ≥ 6.23

[b] Number of ADA-positive subject data tested in the nAb assay

[c] percent nAb-positive subjects calculated using total N in each treatment arm

[d] percent nAb positive subjects calculated as a percentage of ADA-positive subjects tested in the nAb assay

ADA, anti-drug antibodies; nAb, neutralizing antibodies

SUPPLEMENTARY FIGURES

Figure S1. Dose adjustment algorithm (A) and adjusted doses for each treatment group (B)

LDL-C, reduce low-density lipoprotein cholesterol; N/A, not applicable.

A

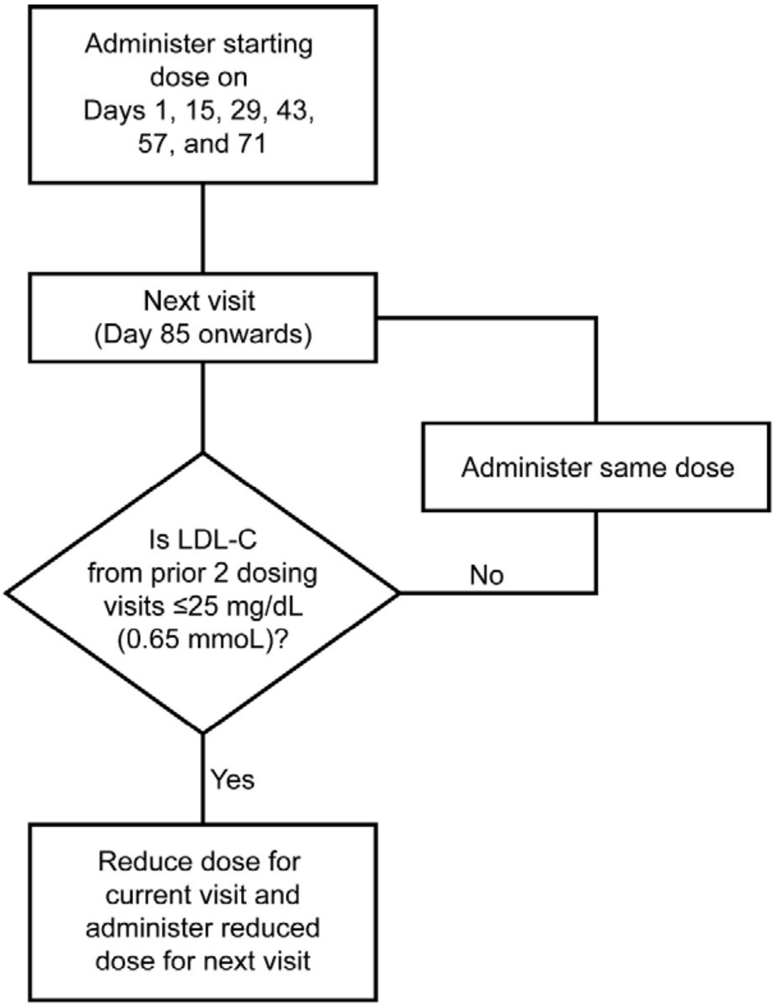

B

Dosing regimen	Dose	Downward dose adjustment
Every 14 days (Q14d)[1]	Placebo	N/A
	Bococizumab 150 mg	75 mg
	Bococizumab 100 mg	50 mg
	Bococizumab 50 mg	Placebo

Figure S2. Mean plasma concentration–time profiles of bococizumab on Day 1 (following a single subcutaneous dose; top panels) and on Day 99 (multiple subcutaneous doses; bottom panels) in atorvastatin-treated (A and C) and treatment-naïve patients (B and D)

Error bars represent standard deviation

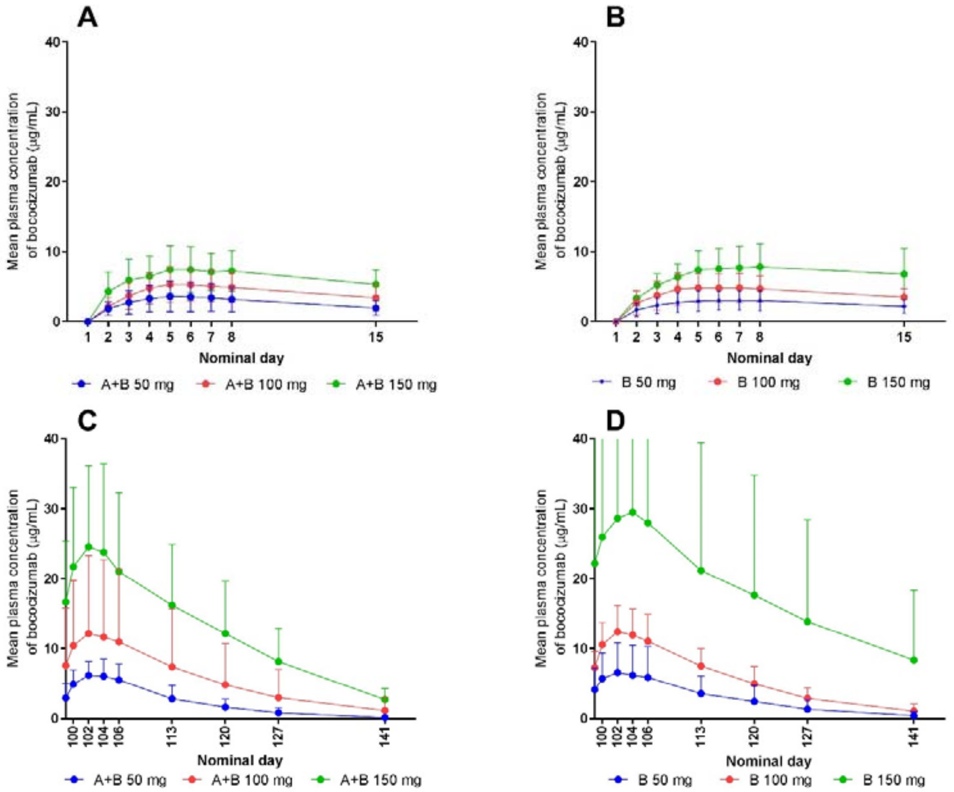

Figure S3. Dose-normalized C_{max} (left panel) and AUC_τ (right panel) for bococizumab (individual and geometric mean plasma values)

AUC_τ, area under the plasma concentration–time curve; C_{max}, maximum plasma concentrations. Open circles represent individual values and filled circles represent geometric means.

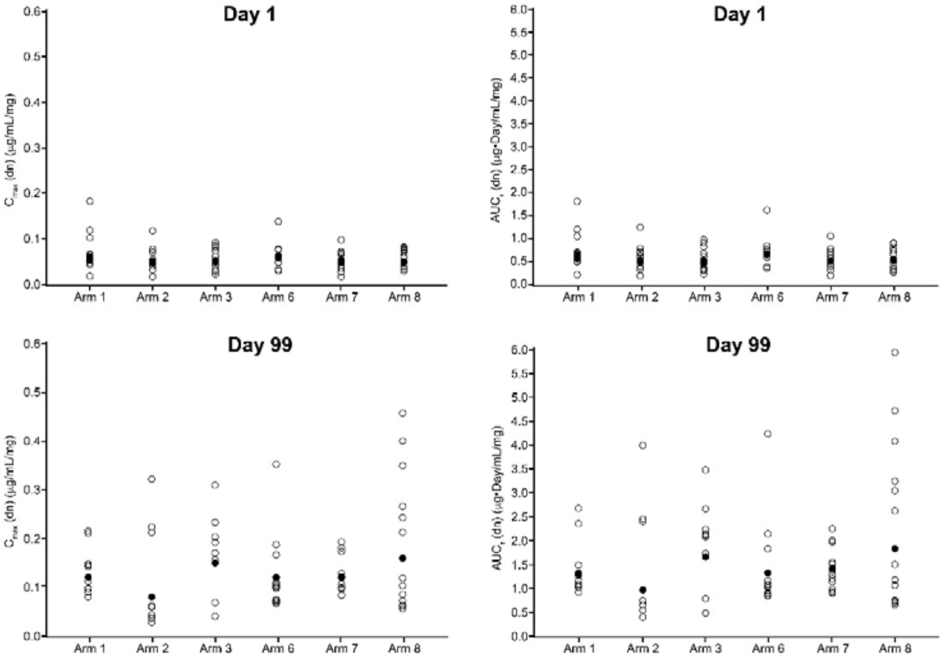

Arm 1 = Atorvastatin + Bococizumab 50 mg, Arm 2 = Atorvastatin + Bococizumab 100 mg
Arm 3 = Atorvastatin + Bococizumab 150 mg, Arm 6 = Bococizumab 50 mg
Arm 7 = Bococizumab 100 mg, Arm 8 = Bococizumab 150 mg

Figure S4. Median trough plasma bococizumab concentration-time profiles for atorvastatin-treated (A) and treatment-naïve patients (B)

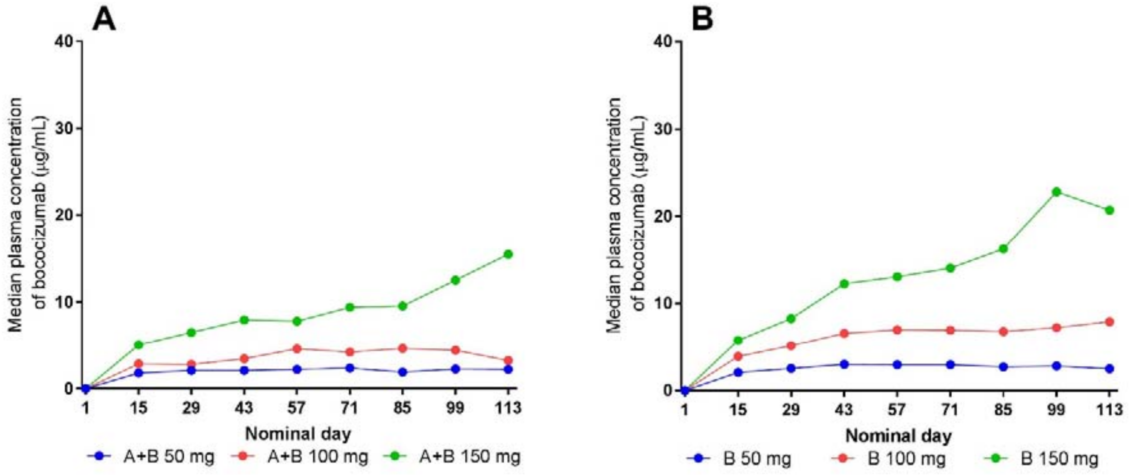

Figure S5. Mean percent change from baseline in plasma PSCK9 concentration for atorvastatin treated (A) and treatment-naïve (B) patients

PCSK9, proprotein convertase subtilisin/kexin type 9. Error bars represent standard deviation

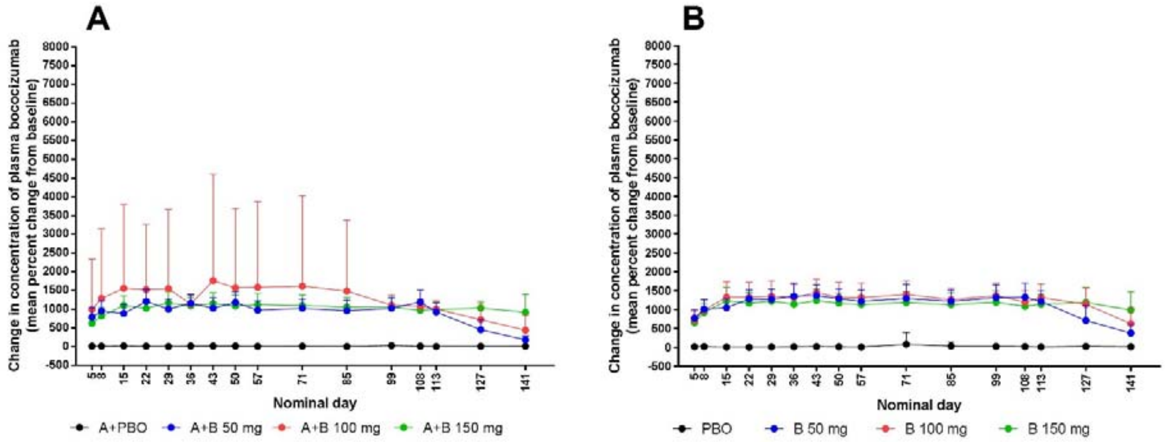

Figure S6. Correlation between LDL particle and Lp(a) concentrations (percent change from baseline) in atorvastatin-treated (A and C) and treatment-naïve (B and D) patients. Figures A and B include treatment groups and placebo while Figures C and D only include the treatment groups.

LDL, low-density lipoprotein; Lp(a) and lipoprotein(a). Each population has a single large outlier that is not shown, to enable the axes to be sized so that the trends in the other values can be easily visualized.

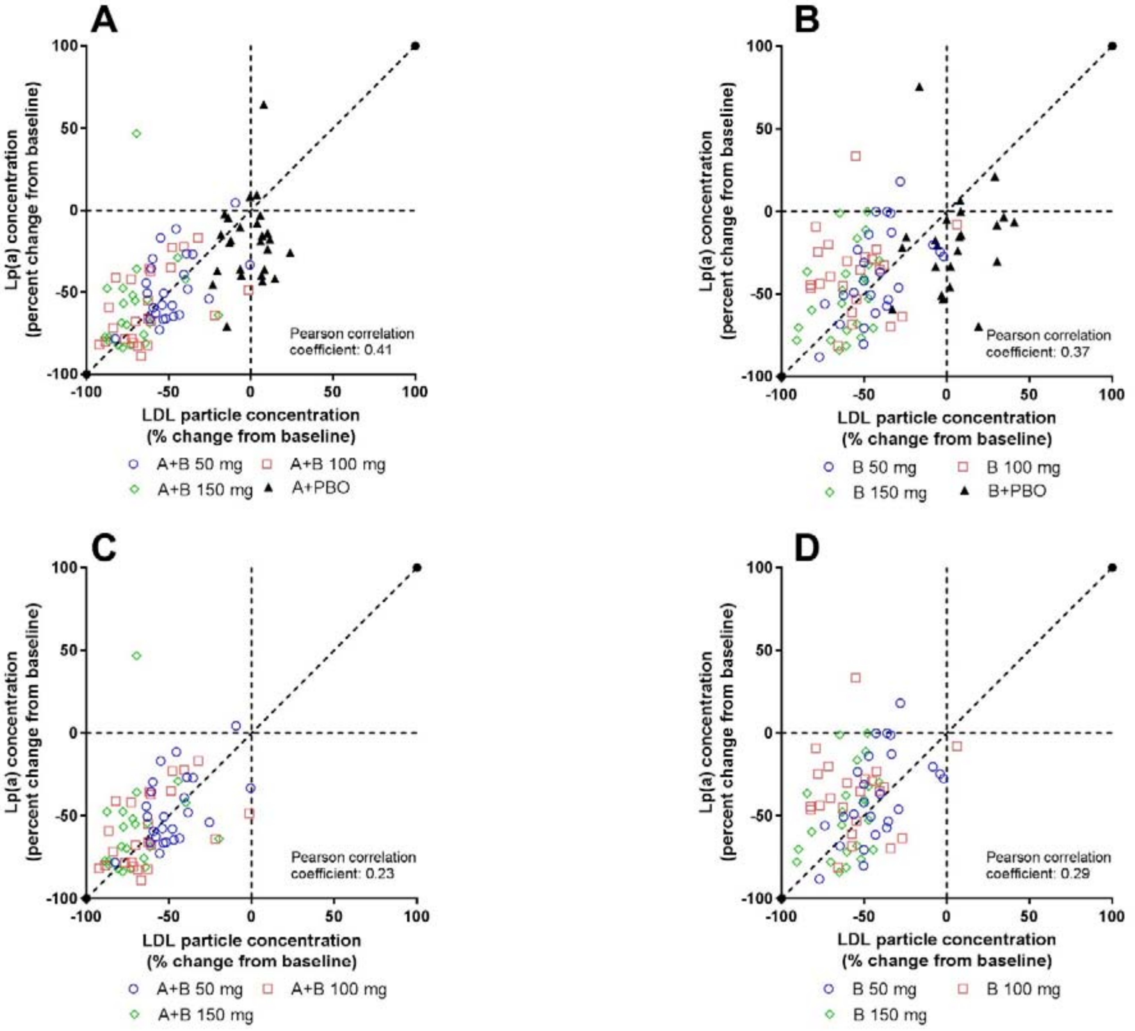

Figure S7. Concentrations of LDL-C (A), PCSK9 (B), bococizumab (C), and anti-drug antibody and neutralising antibody titers for subject 10031047 (atorvastatin-naïve population).

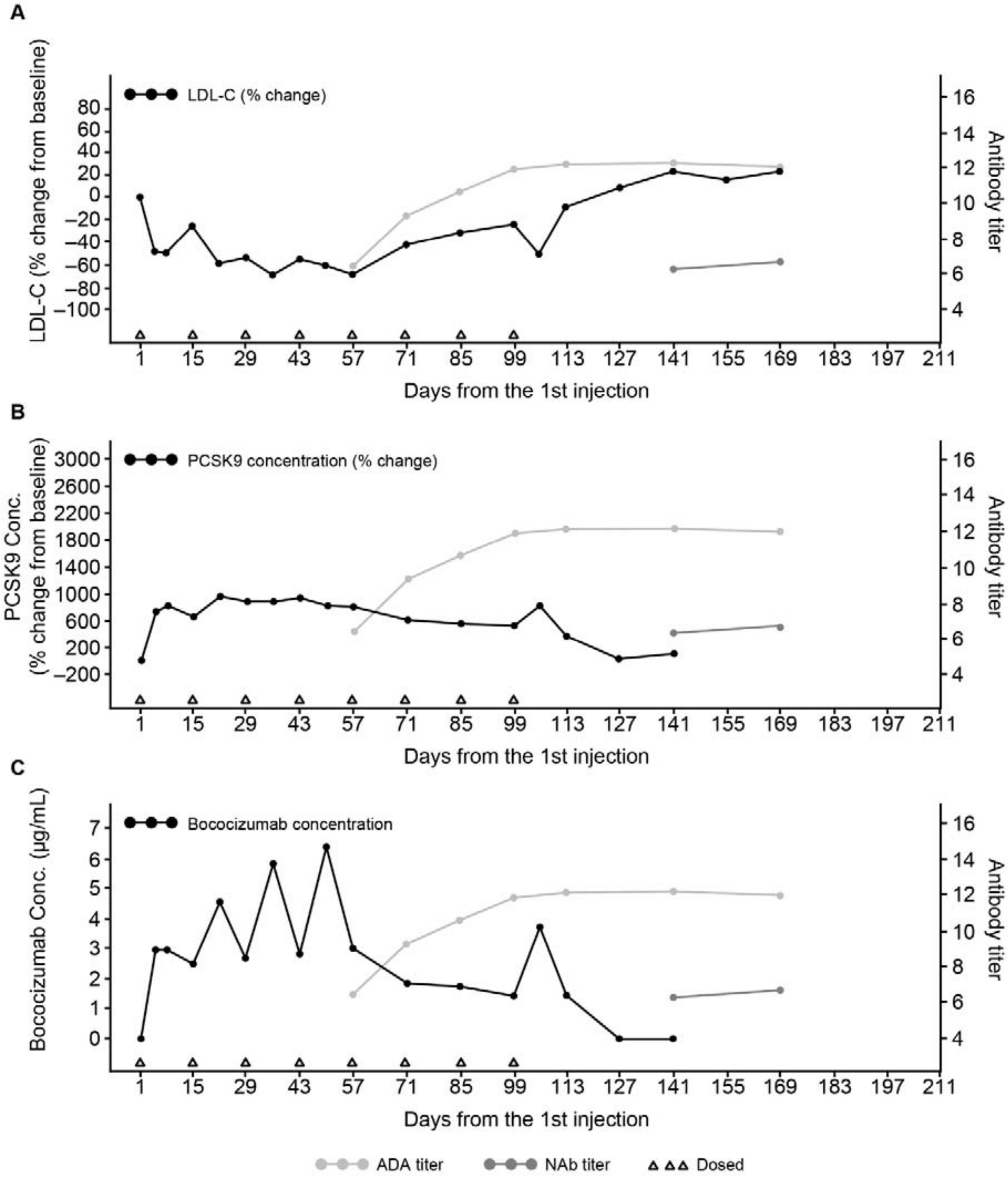

Supplement: Supplemental material [file intjclinpharmacol-57-575-S01.pdf]
